# Synergistic Information Transfer in the Global System of Financial Markets

**DOI:** 10.3390/e22091000

**Published:** 2020-09-08

**Authors:** Tomas Scagliarini, Luca Faes, Daniele Marinazzo, Sebastiano Stramaglia, Rosario N. Mantegna

**Affiliations:** 1 Dipartimento Interateneo di Fisica, Universitá Degli Studi di Bari Aldo Moro, 70126 Bari, Italy; tomas.scagliarini@ba.infn.it; 2 INFN, Sezione di Bari, 70126 Bari, Italy; 3 Dipartimento di Ingegneria, Universitá di Palermo, 90128 Palermo, Italy; luca.faes@unipa.it; 4 Data Analysis Department, Ghent University, 9000 Ghent, Belgium; daniele.marinazzo@ugent.be; 5 Dipartimento di Fisica e Chimica, Universitá di Palermo, 90123 Palermo, Italy; rosario.mantegna@unipa.it; 6 Complexity Science Hub Vienna, 1080 Vienna, Austria; 7 Computer Science Department, University College London, London WC1E 6BT, UK

**Keywords:** synergy, higher order dependencies, financial markets

## Abstract

Uncovering dynamic information flow between stock market indices has been the topic of several studies which exploited the notion of transfer entropy or Granger causality, its linear version. The output of the transfer entropy approach is a directed weighted graph measuring the information about the future state of each target provided by the knowledge of the state of each driving stock market index. In order to go beyond the pairwise description of the information flow, thus looking at higher order informational circuits, here we apply the partial information decomposition to triplets consisting of a pair of driving markets (belonging to America or Europe) and a target market in Asia. Our analysis, on daily data recorded during the years 2000 to 2019, allows the identification of the synergistic information that a pair of drivers carry about the target. By studying the influence of the closing returns of drivers on the subsequent overnight changes of target indexes, we find that (i) Korea, Tokyo, Hong Kong, and Singapore are, in order, the most influenced Asian markets; (ii) US indices SP500 and Russell are the strongest drivers with respect to the bivariate Granger causality; and (iii) concerning higher order effects, pairs of European and American stock market indices play a major role as the most synergetic three-variables circuits. Our results show that the Synergy, a proxy of higher order predictive information flow rooted in information theory, provides details that are complementary to those obtained from bivariate and global Granger causality, and can thus be used to get a better characterization of the global financial system.

## 1. Introduction

Many countries have equity markets. The overall performance of these markets is typically summarized by stock market indices. Economic globalization has interconnected financial markets of different countries. Market movements, and economic and financial news generated or associated with a specific market are almost immediately transmitted to the other markets by professional information providers, media, and social media, making the global financial system highly interconnected. The influence of foreign investment on emerging countries has been investigated thoroughly in [1], and it has been shown that emerging and mature markets are much more integrated today than in the past. The influence among Pacific Rim countries has been explored in [2]. Moreover, it is well known that China and Japan hold huge investments in Brazil. As a result of these interconnections, the log-return dynamics of market indices are correlated among them and fluxes of information are present between market indices. Examples of quantitative estimation of correlations observed between market indices can be found in [3,4,5,6,7,8,9].

A few years after the first studies on correlation of market indices [3,4,5], other studies investigated information flow between these indices by using transfer entropy as a quantitative indicator of the flux. In [10], a causality analysis has shown that higher foreign investment flows can lead to the development of the domestic banking system. Transfer entropy [11] was introduced by Schreiber in 2000, and the first application to finance this information-theoretical measure distinguishing driving and responding elements in subsystems was proposed in [12]. Other investigations of transfer entropy between pairs of market indices or sector indices have been published in [13,14,15,16,17,18,19,20,21]. The investigation of information flows in complex systems has also considered the role of global variables in the process of information flow. In a system with several agents, the so-called global transfer entropy (GTE) averages the information flow to a given agent from all other agents [20,22].

Transfer entropy [22] has deep relations with Granger causality [23]. In fact, the two quantities are strictly proportional when the underlying stochastic processes are Gaussian [24]. Granger causality has also been used to detect directed networks of elements starting from the analysis of their time series. A first influential paper extracting a directed network from the statistical analysis of a financial multivariate time series on the basis of Granger causality tests was reference [25]. Since then, Granger causality has also been used to investigate Granger causation between pairs of stock market indices [26,27,28].

Pairwise interdependencies may not fully describe the complexity of information flows. Higher-order multivariate effects may play a role in the global financial systems. Information theory, which is commonly used to quantify interactions between two variables, has recently been extended to provide multivariate information measures that have been increasingly used to investigate interactions between groups of three or more variables, often with an emphasis on the so-called synergistic and redundant interactions. While bivariate information measures are commonly agreed upon, the multivariate information measures are still the topic of active research, see, e.g., in [22]. In this context, redundancy and synergy are intuitive yet elusive concepts, with definitions that range from the purely operative to the most conceptual ones, see in [29,30] and the references therein. When analyzing interactions in multivariate time series, redundancy may arise if some channels are all influenced by another signal which is not included in the regression; another source of redundancy may be the emergence of synchronization in a subgroup of variables, without the need of an external influence. Redundancy manifests itself through a high degree of correlation in a group of variables, both for instantaneous and lagged influences. A complementary concept to redundancy is synergy, corresponding to variables which provide more information when treated jointly [31]. Redundancy and synergy have been further connected to information transfer in [32], where an expansion of the information flow has been proposed, and in the partial information decomposition (PID) introduced in [33], where a multiscale approach has been proposed. Recently, it has been demonstrated that the physical quantity that reveals an upcoming transition, at least in the two dimensional Ising model, is actually the synergy, which peaks in the disordered phase, while the redundancy reaches its maximum at the critical temperature [34]. It is remarkable that while the evaluation of GTE requires the knowledge of all the variables, PID can be applied to just three variables: a triplet of interacting variables can thus be seen as a proxy of the critical behavior of the whole system. The PID framework has been applied to the cardiovascular and respiratory systems in [35], to fMRI data in [36], and to the analysis of the muscular system in [37].

The purpose of this work is the application of PID to a set of financial market indices in order to characterize the market dynamics in terms of the level of predictability of each given market exploiting three-body interactions and the corresponding synergy, so as to go beyond the description of the causality pattern based only on pairwise influences. Specifically, we analyze the information flow among market indices of financial markets located in Europe, America, and Asia. We focus on the information flow originating in the European and American markets and impacting on Asian financial markets.

## 2. Data

We consider seventeen stock market indices that belong to three groups: 4 indices of American stock markets (labeled as AM), 7 indices of European stock markets (labeled as EU), and 6 indices of Asian stock markets (labeled as AS). In particular, the indices of American stock markets are S&P500, Russell 2000, Ibovespa index (Brazil), and TSX Composite Index (Toronto). The indices of European stock markets are German DAX (Frankfurt), FTSE 100 (London), CAC40 (Paris), BFX (Brussels), Italian FTSE MIB, Spanish IBEX3,5 and Swiss Market Index SMI. The third group consists of stock market indices Nikkei 225 (Tokyo), Hang Seng HSI (Hong Kong), SSE Composite Index (Shanghai), KOSPI 200 Composite Stock Price Index (Korea), BSE Sensex Index (Bombay), and FTSE Straits Times Index (Singapore). Daily openings and closing prices from 1 January 2000 to 31 December 2019 have been collected from Quandl [38] and Yahoo Finance [39]. During the investigated years, several financial crises occurred. It is worth mentioning (i) the crash of the dotcom bubble, whose bubble burst lasted from March 2000 to October 2002, with effects until the beginning of 2003; (ii) the Global Financial Crisis of 2007–2009, which had such a global impact as it spread over most of the countries like an unstoppable domino; (iii) the European sovereign debt crisis, started in correspondence of the August 2011 stock markets fall, when the European stock markets suffered heavy losses due to fears about the world economic outlook; and (iv) the Chinese stock markets turbulence in 2015–2016. As so many events occurred, each with its own peculiarities, we decide to adopt a window approach, selecting non-overlapping windows. Varying the width of the time windows, we realize that the synergistic information flow appears to be localized in time rather than being a continuous exchange of information. However, application of PID requires a suitable number of samples, therefore a proper localization of the events (when synergistic dependencies occur) is unfeasible; indeed, in order to have statistical reliability of results, the window cannot be too small. In this paper, we show the results for windows corresponding to one calendar year, a conventional and easily interpretable duration, and leave to further research the development of methods to deal locally in time the issue of synergistic information flow.

Denoting $p_{i}^{C}\left(t\right)$ the closing price of the *i*-th stock market index on day *t*, daily logarithmic returns are calculated for every market index as

(1) $$x_{i}\left(t\right)=\log\left(p_{i}^{C}\left(t\right)\right)-\log\left(p_{i}^{C}\left(t-1\right)\right).$$

The same procedure is applied to the opening price for the i-th stock market index $p_{i}^{O}\left(t\right)$ to obtain the overnight change

(2) $$y_{i}\left(t\right)=\log\left(p_{i}^{O}\left(t\right)\right)-\log\left(p_{i}^{C}\left(t-1\right)\right).$$

We verify that both *x* and *y* variables can be treated as stationary variables by performing an Augmented Dickey-Fuller test. The property of stationarity is a necessary condition for the information theoretical analyses that we apply in this work.

In this type of study, it is very important to properly take into account the time zone effect [26]. The selected stock markets operate in different time zones and the opening and closing times of markets differ accordingly. In order to avoid the bias due to the time zone effect, in this paper we analyze only the information flowing in circuits made of three markets, where the target belongs to the AS group and two drivers belong to AM and/or EU groups. Moreover, we concentrate on the prediction of the overnight change of asiatic markets based on the knowledge of European and American markets closing prices at the day before. This choice ensures that the target variable cannot receive information from the driving variables in the same day. Consequently, we label stock market indices of the AS group as the y(t) time series, while markets in AM and EU groups are associated with the x(t) time series. In other words, we study the predictive information flow in pairwise directed interactions $x_{\alpha }\to y_{\gamma }$ and triplet circuits $\left\{x_{\alpha },x_{\beta }\right\}\to y_{\gamma }$, where α and β are in AM or EU groups, and γ is in AS group. It is worth mentioning that due to the timing of markets openings, the same analysis would not be possible for circuits with drivers in Asia and Europe and the target being American, indeed the European markets close when American markets are already open, therefore the informational character of such triplets would not be comparable with those of circuits America-Europe → Asia. Particular care has been spent to cope with the problem of missing records arising, e.g., when stock markets are closed in some countries due to national holidays. To cope with this, for each triplet of stock market indices, the samples for the estimation of causalities have been constructed taking just the days where data of all the three indexes were available as well as records of the following day.

## 3. Methods

In the next sections, we provide details of the adopted prediction measures and statistical methodology.

### 3.1. Bivariate Granger Causality

Let us consider the overnight change series of the *i*-th stock market index, $y_{i}$, as the target variable (i=1,…,m), and the daily return series of the *j*-th stock market index, $x_{j}$, as the driver variable (j=1,…,n) measured in a given time window; in this work, m=6 is the number of AS markets and $n=n_{1}+n_{2}$, where $n_{1}=4$ AM markets and $n_{2}=7$ EU markets are considered. Then, calling $\epsilon (y_{i}|Y_{i})$ the mean squared error prediction of $y_{i}\left(t\right)$ on the basis of its past states $Y_{i}\left(t\right)=\left\{y_{i}\left(t-1\right),y_{i}\left(t-2\right),\;\ldots ,\;y_{i}\left(t-l\right)\right\}$, and $\epsilon (y_{i}|Y_{i},X_{j})$ the mean squared error prediction on the basis of both $Y_{i}\left(t\right)$ and $X_{j}\left(t\right)=\left\{x_{j}\left(t-1\right),x_{j}\left(t-2\right),\;\ldots ,\;x_{j}\left(t-l\right)\right\}$, the bivariate Granger causality (GC) is defined as the following statistics [40],

(3) $$G_{j\to i}=\log\epsilon (y_{i}|Y_{i})-\log\epsilon (y_{i}|Y_{i},X_{j}).$$

Repeating this evaluation for each i∈{1,…,m} and j∈{1,…,n}, we obtain the pattern of bivariate causality from any AM/EU stock market index to any AS index in the given window.

### 3.2. Global Granger Causality

In the present study, we consider an overall measure of predictive information transfer between two groups of variables, see in [22], computing the global Granger causality (GGC) from European and American markets to the Asian market as follows,

(4) $$G^{\left(gl\right)}=\frac{1}{m}\sum_{i=1}^{m}G_{i}=\frac{1}{m}\sum_{i=1}^{m}\left(\log\epsilon (y_{i}|Y_{i})-\log\epsilon (y_{i}|Y_{i},X)\right),$$

where $\epsilon (y_{i}|Y_{i},X)$ is the mean squared error prediction of $y_{i}\left(t\right)$ on the basis of both its past states $Y_{i}\left(t\right)$ and the past states of all the variables related to AM/EU stock market indices, collected in the vector $X(t)=[X_{1}\left(t\right),\;\ldots ,\;X_{n}\left(t\right)]$. For each AS stock market index $y_{i}$, $G_{i}$ measures the information provided by all the AM/EU stock market indices $\left\{x_{1},\;\ldots ,\;x_{n}\right\}$ about the future value of $y_{i}$; the result is then averaged over the *m* AS indexes to get the global measure.

As far as the order of the model is concerned, we fix l=1, as we are interested here in the immediate influence, namely, in how the present record influences the state of the next record. Because of the high efficiency of information spreading in financial systems, and due to the stylized fact that the autocorrelation of the index return vanishes in a very short period of time, the choice l=1 is robust against spurious causality due to longer memory effects. A similar choice has been adopted in several studies dealing with transfer entropy, Granger causality and global transfer entropy [12,14,16,18,19,21,27].

In order to evaluate the square prediction errors leading to the Granger causality measures, we use linear models. Moreover, to assess the statistical validity of the GGC, we estimate its value expected under the null hypothesis of independence by using surrogate random time series of the target stock market indices obtained with the method described in [41]. Specifically, we generate surrogate data of the target time series by using the Iterative Amplitude Adapted Fourier Transform (IAAFT) algorithm of Schreiber and Schmitz [42], and we consider the empirical GGC value compatible with zero when we cannot reject the hypothesis that such value is generated by a randomized version of the empirical data. The threshold for statistical significance used in our tests is 0.05. The present validation procedure is the most common in the Granger causality literature, and requires stationarity of processes. However, other choices are possible, e.g., bootstrap.

### 3.3. Partial Information Decomposition

The partial information decomposition (PID) is obtained starting from the GC from a pair of drivers, comparing these values with the GC from single drivers as detailed in [33]. Hereafter, we briefly recall the approach. The GC from the pair of stock market indices $x_{j}$ and $x_{k}$ to the target stock market index $y_{i}$ (j,k∈{1,…,n},j≠k;i∈{1,…,m}) is defined as

(5) $$G_{jk\to i}=\log\epsilon (y_{i}|Y_{i})-\log\epsilon (y_{i}|Y_{i},X_{j},X_{k}).$$

The information decomposition is defined as

$$\begin{array}{c} G_{jk\to i}=U_{j\to i}+U_{k\to i}+R_{jk\to i}+S_{jk\to i},\\ G_{j\to i}=U_{j\to i}+R_{jk\to i},\\ G_{k\to i}=U_{k\to i}+R_{jk\to i}, \end{array}$$

where the pairwise GC $G_{k\to i}$ is given by (3) and a similar expression holds for $G_{k\to i}$.

In the above definitions, the terms $U_{j,i}$ and $U_{k,i}$ quantify the components of the information about the target $y_{i}$ which are unique to the sources $x_{j}$ and $x_{k}$, respectively, thus reflecting contributions to the predictability of the target that can be obtained from one of the sources when it is treated as the only driver, and not from the other source. Each of these unique contributions sums up with the redundant information $R_{jk\to i}$ to yield the information transfer between one source and the target according to the classic Shannon information theory. The term $S_{jk\to i}$ is called Synergy and refers to the ability of the two sources to provide additional information about the target when they are considered jointly as information sources. In other words, it is the information that is uniquely obtained by using the two sources $x_{j}$ and $x_{k}$ together, but not considering them alone. As, in the above definitions, four quantities are unknown and just three equations are at hand, the information decomposition in unique, redundant, and synergistic parts is a missing piece in classical information theory. To obtain the Synergy measure $S_{jk\to i}$, we adopt the prescription of [43], and take as the Redundancy $R_{jk\to i}$ the minimum between the two pairwise Granger causality indices $G_{j\to i}$ and $G_{k\to i}$.

Furthermore, in PID analysis, in order to assess the statistical significance of the empirical values of Synergy, we generate surrogates of the target time series by the IAAFT algorithm [42], and we consider compatible with a zero value those values of the Synergy for which the null hypothesis of uncoupled processes is not rejected at the 0.05 statistical threshold.

## 4. Results

### 4.1. Pairwise and Global Granger Causality

**Figure 1:**
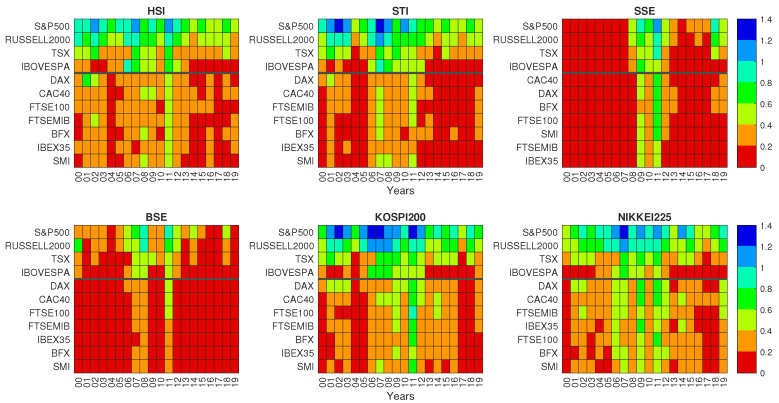
Granger Causality at different calendar years. Each panel refers to a different Asian stock market index target. In each panel, the Granger-causing stock market indices (each one associated with a row) are divided into 2 continental groups (separated by an horizontal thick black line). The top group comprises American stock market indices and the bottom group European stock market indices. Within each group, the Granger-causing stock market indices are ordered by the GC averaged on all years.

We start considering the pairwise GC of the data set, with the aim of finding those stock market indices with the strongest influence on the group of Asian stock market indices, as well as the most influenced Asian stock market index. In Figure 1, the GC values obtained for each calendar year are shown for all pairs of driver and target stock market index, showing that the pairwise influence is observed over an extended period of time for KOSPI 200, Hang Seng, Nikkei 225, and Straits Times, whereas high values of GC are localized in specific years for SSE Composite and BSE Sensex indices.

Figure 1 shows that highest values of GC are observed for SP500 and Russell 2000 indices among American stock market indices and for DAX and CAC40 among European stock market indices. The figure also shows a pronounced time dynamics with highest values of GC clustering during the years 2007–2009 and 2011–2012. As we have already noticed, the time dependence of GC in the six target markets divides them in two groups. The first group is the group of KOSPI 200, Hang Seng, Nikkei 225, and Straits Times indices and the second group comprises SSE Composite and BSE Sensex indices. The first group has associated a constant flow of information from the American and European stock market indices whereas for the second group we detect sizable flow of information only during period of worldwide financial distress.

We also compute the Global Granger causality from the 11 American and European stock market indices and each of the Asian stock market indices. In Table 1, we summarize the values of GGC for each target Asian stock market index as a function of the calendar year. The GGC results are similar to the results obtained for the pairwise GC. In fact, GGC from American and European stock market indices is detected for all calendar years for KOSPI 200, Hang Seng, Nikkei 225, and Straits Times indices, whereas for the SSE Composite and BSE Sensex, the estimated GGC values are lower than for the other indices, and for some years they are so low that they turn out to be compatible with the one observed for a randomized version of the target (in this case they are not reported in the Table). In summary, also this measure shows that the Shanghai Stock Exchange and the Bombay Stock Exchange are less effected than the other considered Asian stock market indices from the performances of the selected American and European stock market indices.

### 4.2. Synergy

**Figure 2:**
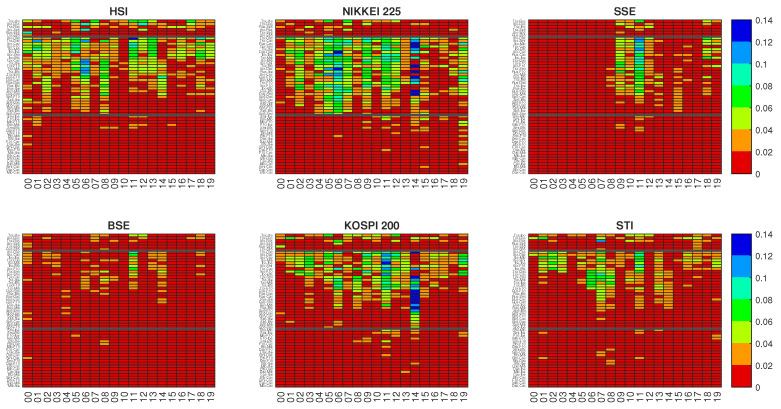
The Synergy *S* for each calendar year. Each panel represents an Asian stock market index target. The 55 triplets of stock market indices in each panel are divided into 3 groups (from top to bottom separated by a black thick line): the first group includes both driving stock market indices from the American continent (6 triplets originating from 4 American stock market indices and the Asian target), the second group includes one driving stock market index from the American continent and the other from the European continent (28 triplets originating from 4 American stock market indices, 7 European stock market indices, and the Asian target), and the third group includes both driving stock market indices from the European continent (21 triplets originating from 7 European stock market indices, and the Asian target). Within each group triplets are ordered according to the average value of Synergy averaged on all years. In each panel, for the sake of simplicity the driving stock market indices are all labeled with a three letters code. The American stock market indices are labeled as **SP5** for S&P500, **Rus** for Russel 2000, **Ibo** for IBOVESPA and **Tsx** for TSX. The European stock market indices are labeled as **Dax** for DAX, **Bfx** for BFX, **Mib** for FTSE MIB, **F10** for FTSE 100, **Cac** for CAC 40, **Ibe** for IBEX 35, and **Smi** for SMI.

In this section, we present our results about the Synergy associated with pairs of stock market indices located in America and Europe when they are used to predict the overnight return of some Asian stock market indices. In Figure 2, for each Asian stock market index considered as a target, we show the value of the Synergy for all possible n(n−1)/2= 55 triplets of of stock market indices involving the Asian Target and the n= 11 European and American stock market indices.

By analyzing Figure 2, we notice that the triplets with highest values of Synergy are those where the two drivers are one American and one European (i.e., in triplets of the second group of the figure). High values of the Synergy are also observed in triplets with two American drivers for some specific years. Negligible Synergy values are observed for pairs of drivers consisting of two European stock market indexes (third group of Figure 2).

The figure also shows that there is a pronounced temporal dynamics of the Synergy. Specific targets present high values for specific years. For example when Nikkei 225 and KOSPI 200 are the targets high values of the Synergy are observed in 2014. Another example is provided by SSE Composite and BSE Sensex targets where high values of the Synergy are observed in 2011. Moreover, also for this metrics we note that the Synergy for SSE Composite target appears to be strongly localized in time during the years 2009–2011, whereas for the BSE Sensex target index Synergy values are quite low for all investigated years.

Moreover, for this metric we evaluate with a statistical test whether the measured Synergy is statistically distinct from zero (in these tests we again use 0.05 as a statistical threshold). When the test rejects the null hypothesis that the estimated Synergy is compatible with the one obtained by using a randomized target, we call the time window used to compute the Synergy a validated window (i.e., a time window where the estimated Synergy is statistically distinct from a value obtained with a randomized target).

**Figure 3:**
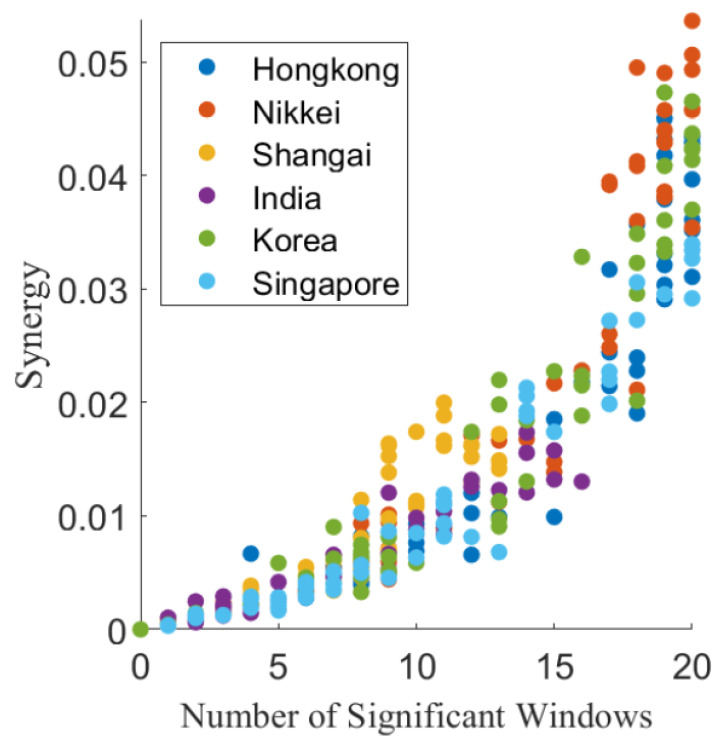
Scatter plot of the average Synergy associated with each triplet of stock market indices averaged over all 20 time windows as a function of the number of validated windows. The color of dots is chosen according to the target stock market index as indicated in the legend box.

In Figure 3, we show a scatter plot of the average Synergy associated with each triplet of stock market indices averaged over all 20 time windows as a function of the number of validated windows. The panel shows that the average Synergy has an approximately quadratic relation with the number of validated windows, suggesting that the triplets whose synergistic influence occurs for more years are also those characterized by highest values of the synergy. In the scatter plot, the color of dots is chosen according to the target stock market index.

**Figure 4:**
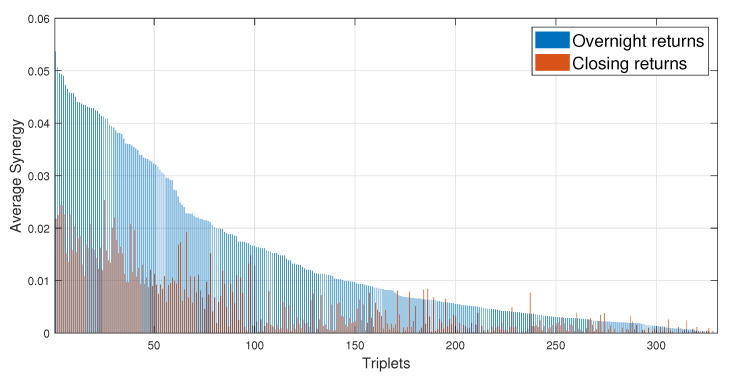
Average Synergy for opening or closing returns of the targets. The bar plot compares the average Synergy estimated by using overnight returns (blue bars) and closing logarithmic price returns (red bars) for the target stock market index. The average Synergy is shown for all 330 triplets as a function of the rank of each triplet. The rank is determined by considering the value of the average Synergy for the overnight returns. The same rank is used also when showing the average Synergy of the closing return.

All the results shown so far refer to the overnight return (difference between the logarithm of the target index at the opening minus the logarithm of the target index at the closing of the previous day). We have also computed the Synergy for the daily return of the target index (i.e., difference between the logarithm of the target index at the closing minus the logarithm of the target index at the closing of the previous day); this was obtained using for the Asian stock market indexes the variables $x_{i}$ and $X_{i}$ in place of $y_{i}$ and $Y_{i}$ in Equations (3) and (5) during PID analysis. The results obtained for all 330 triplets are shown in Figure 4. The figure shows the average Synergy of each triplet both when the target is the overnight return of the Asian stock market index (blue bars in the figure) and when the target is the daily return (close to close labeled as red bars in the figure). The average Synergy for the overnight returns is larger than the average Synergy for the daily return for the large majority of triplets. In fact only a few exceptions are observed and they occur for low values of the average Synergy. This observation suggests that the information associated with the closing price of European and American stock markets is incorporated into the price dynamics of the Asian stock market indices immediately after the opening of the Asian markets.

## 5. Discussion and Conclusions

Use of causality analysis of stock market index returns in the description of the information flow occurring in the global financial system has received growing attention during the last years. In the present work, we provide the first study of the information flow detected among groups of three stock market indices over a period of twenty years. Our analysis is performed by investigating the so-called Synergy, an information theoretical measure that has been recently introduced to account for multivariate interaction effects in causality analysis.

The global financial system is operating worldwide in all continents. For this reason, the activity of different markets is scheduled at different time intervals due to the presence of different time zones. To investigate information flows compatible with the sequence of market activities occurring worldwide in a trading day, we consider information flow that has targets in Asian markets and driving signals in previous European and American markets.

Moreover, in the regression models we choose to focus on a specific form of information flow. We consider the driving signals as originated by the closing returns (close to close daily return) of European and American stock market indices, and we consider as target signal the subsequent overnight change of Asian stock market return (open to close daily return). To our knowledge, this is the first time this choice is adopted in a causality analysis of stock market indices. Our results show that predicting the open to index return leads to higher causality metrics with respect to those that one would obtain predicting the close to close returns, see Figure 4. We interpret this result as an evidence that markets digest quite quickly the information flow originated in stock markets of other countries.

In addition to the Synergy investigation, we also estimated bivariate GC and GTE between driving and target indices. Concerning bivariate GC analysis, we find that the most important sources of information are the US indices SP500 and Russell 2000, whereas the most influenced Asian stock market indices are KOSPI 200, NIKKEI 225, HSI, and STI (especially from American stock market indices). For these indices, the information flow is detected for all years. The information flow of European stock market indices is less pronounced and more localized in time especially during the years of the financial crisis originated in 2007–2008 and turned out into sovereign debt crisis into 2011–2012. This years of crisis are also the years when the information flow is observed for SSE Composite Index and BSE Sensex Index. A similar temporal pattern is observed for the GTE with highest values of this metrics observed during the years 2007–2012.

Coming back to Synergy results, it is worth noting that the highest values of Synergy are observed when the two stock market index drivers involve an European and an American stock market index (see Figure 2).

Moreover, Synergy seems more relevant when a middle size American market is involved. In fact, the highest values of the Synergy are observed when driving indices include IBOVESPA or TSX, although their influence is rather low with respect to SP500 and Russell 2000 in the bivariate GC analysis. It is well known that both China and Japan hold huge investments in Brazil, and our analysis suggests that information about the Brazil main stock market index is informative for HSI and NIKKEI 225, jointly with information from other European stock market indices.

Our results thus show that the Synergy, i.e., a proxy of higher order information flow rooted in information theory, provides details that are complementary to those obtained from the bivariate and global GC analysis, and can thus be used to get a better characterization of the global financial system.

In order to better characterize higher order dependencies of global financial market, further research will be devoted to develop methodologies capable to estimate locally in time the synergistic information flow, indeed the synergistic information flow appears to have a localized nature rather than resembling a nearly continuous exchange of information.

## Figures and Tables

**Table 1:** Global Granger causality on Asian stock market indices. Table shows $G_{i}$ (as defined in (4)) for each calendar year. For each Asian stock market index target, the GGC is computed by using the 11 American and European stock market indices investigated in this paper. The values in parenthesis represent the 5 and the 95 percentile of the GGC computed for the IAAFT surrogates. Values labeled with an asterisk are compatible with the values obtained for surrogate data. When this occurs we say that the estimation of the variable is not statistically validated in the considered time window.

	SSE	KOSPI 200	BSE	HSI	Nikkei 225	STI
2000	0.17*0.190.04	0.830.210.05	0.760.170.05	1.500.210.04	0.860.180.05	1.370.200.04
2001	0.11*0.230.03	1.600.200.04	0.440.170.04	1.580.210.05	1.080.180.04	1.750.180.04
2002	0.14*0.190.03	1.760.190.04	0.610.180.04	1.840.150.04	1.430.190.04	1.810.160.04
2003	0.08*0.160.03	1.250.190.04	0.580.160.03	1.210.170.04	1.130.160.03	1.390.170.04
2004	0.09*0.190.04	0.850.190.04	0.330.180.04	1.120.190.04	1.310.160.05	0.850.190.03
2005	0.08*0.150.04	1.670.180.04	0.530.160.05	1.530.160.04	1.560.160.03	1.220.180.04
2006	0.230.180.05	2.120.210.04	0.860.200.05	1.760.220.05	1.950.200.05	1.460.200.04
2007	0.300.200.04	1.770.190.04	0.960.190.04	1.680.190.04	1.870.190.04	2.120.200.05
2008	0.800.210.04	2.000.200.05	1.600.180.04	1.520.200.04	1.350.200.05	1.820.200.05
2009	1.560.190.03	1.530.180.04	1.000.160.03	1.180.180.04	1.480.190.04	1.250.160.05
2010	1.110.170.03	1.530.150.04	0.530.170.04	1.110.150.04	1.500.160.04	1.560.170.04
2011	2.050.170.04	1.940.190.05	1.360.200.04	1.940.210.04	1.540.220.06	1.760.180.05
2012	0.790.200.05	1.630.230.05	0.640.200.05	1.450.190.05	1.490.230.04	1.000.200.05
2013	0.600.200.04	1.160.210.06	0.490.190.05	1.260.220.05	0.980.230.05	1.100.190.05
2014	0.270.190.04	1.750.170.04	1.160.200.05	0.980.180.05	2.060.220.05	0.820.190.05
2015	0.440.190.04	1.200.170.04	0.660.180.03	1.190.160.04	1.640.170.05	1.310.190.04
2016	0.460.220.04	1.320.180.04	0.17*0.210.04	0.760.190.05	0.840.190.05	0.830.200.04
2017	0.500.200.06	1.020.180.05	0.280.190.05	1.180.220.05	0.870.200.05	0.980.190.05
2018	1.770.190.05	1.620.200.05	0.920.210.05	1.410.190.05	1.270.190.04	1.300.190.04
2019	0.670.160.04	0.980.180.05	0.310.210.04	0.910.200.05	1.370.210.04	0.850.220.06
